# The Asylum Holocaust

**Published:** 1903-01-31

**Authors:** 


### HOSPITAL ADMINISTRATION.

### CONSTRUCTION AND ECONOMICS.

### THE ASYLUM HOLOCAUST.

A FEW PRACTICAL QUESTIONS.

The whole country has been shocked, as well it may be, by the fact that upwards of fifty female pauper lunatics have been done to death by fire at Colney Hatch Lunatic Asylum. It would be wrong to express at this moment any opinion as to the responsibility attaching to the Lunacy Commissioners, the London County Council and its Asylum Committee, and those immediately charged with the duty of managing and controlling the establishment at Colney Hatch. In regard to the Colney Hatch fire it is necessary in the public interest to ask a few plain questions. From the report in the *Times*, it appears that some 300 female lunatics and 40 members of the asylum staff had been housed for seven years in a row of temporary structures—huts of corrugated iron and wood occupying an area of about two acres. There were five pavilions in all, each of which consisted of a day-room and dormitory on opposite sides of a central corridor. Three of the five wards were used for infirmary patients and two for chronics, all being female pauper lunatics. The infirmary wards were situated nearest to the main buildings at a point most distant from the spot where the fire originated, and all these dependent patients appear to have been rescued.

That is an important fact to bear in mind as exhibiting the time available for rescue of the patients. Those who perished are stated to have been inmates of the pavilions set apart for "the chronics," *i.e.*, presumably the able-bodied pauper lunatics. To any trained administrator who has a knowledge of asylums and hospitals, in view of these particulars, the main causes of the disaster may be appreciated from the following facts, which we take from the *Times* report, the medical superintendent's report, and official documents.

The *Times* statement:—"When it was found impossible to remain in one dormitory any longer, the officers and nurses ran to the next. The doors, *which were of course locked*, were battered in." (Who had the keys ?) "They" (the patients) "were all attired only in their nightdresses, and were terrified by the noises of the fire."

Dr. Seward, the medical superintendent, reports that the fire was discovered by the night nurse on duty and by the stoker in the adjoining boiler-house, who both noticed smoke and flames coming out of the storeroom for patients' clothing. [This storeroom is situated in front of the boiler house near the centre of the first pavilion. It is important to note that the fire did not actually commence in a ward containing patients.] The asylum fire brigade consists of 16 men, nine living in the asylum and seven outside, without any fire engine, steam pumps for pumping the water directly from the main being the appliances in use at Colney Hatch. The water supply is usually taken from a well, but it was undergoing repair, and so the Barnet Company's water had to be used. All those who perished were pauper patients, whilst all the members of the staff (40) escaped.

**Official Facts.**

Colney Hatch is a county asylum and under the control of the London County Council. Its nominal capacity is for 2,488, but at the date of our last return upwards of 3,000 patients were in residence. In addition to other officers there are seven medical officers, 103 male and 183 female attendants, giving a total of 293, so that the staff available was nearly equal to the whole number of patients which had to be removed from the temporary blocks.

**A Few Practical Questions.**

It is essential that precise answers should be forthcoming to the following questions :—

(1) Did the fire originate in the clothes store ? seeing that Captain Wells reports the boiler house was burnt, although it stands some distance in front of the store, so that to reach it the fire must have spread backwards against the wind.

(2) Why is it that the asylum fire brigade consisted of only 16 members, when there were 103 male attendants, in addition to the large number of males who must have been employed in various capacities about the place, and 183 female attendants ?

(3) Is it to be understood that the whole staff, or the majority of them, had never been trained to fire drill, and that this important and usual practice in asylums did not prevail at Colney Hatch ?

(4) Why was it that immediately the alarm of fire was given every door in each pavilion was not immediately unlocked and propped open, so as to afford the maximum of opportunity to the inmates to effect their escape from the burning buildings ? This is a very important question, seeing that the patients burnt are stated to have been "chronics," and so able to readily escape from the buildings if given equal opportunities with the staff, who all got away safely.

(5) In regard to the water supply, were the local brigade justified in "complaining bitterly of the want of water," or is Dr. Seward correct in stating that there was an abundance of water ?

(6) What amount of time elapsed between the discovery of the fire by the night nurse and the stoker and the time when the first ward block was wholly enveloped in flames ?

(7) Did the night nurse and the attendants in the first block attacked immediately unlock and open all the doors, including those to the grounds, and if not, why not ?

(8) Immediately the alarm was given were the attendants and staff assembled, divided into relays under responsible officers, and each relay charged with a definite duty in regard to the inmates and the fire ?

(9) How is it that the whole of the 40 members of the staff were able to escape unhurt from the burning buildings, and that upwards of 50 patients perished ?

These questions should be pressed home, and we have no doubt they will be pressed home by the representatives of the County Council, for that body is placed in a very unenviable position through this calamity. The County Council has for years criticised and harassed everybody who owns or has charge of the administration of any public or important buildings within its jurisdiction, and at the present time it is promoting a fire Bill in Parliament which, if passed in its present form, will, it is stated, impose an expenditure estimated at at least six million pounds upon the owners of buildings and house property within the Metropolitan area. As matters stand at present it would appear as if the energies of the members of the County Council were so absorbed in criticism of other people's methods and conduct that they have failed altogether to realise their own responsibilities, or to adopt careful provisions against fire in regard to the buildings under their charge, though such provisions may be met with everywhere throughout London to-day in regard to great buildings managed by responsible corporations under private management. What the County Council seems to lack is administrative ability. It is so easy to be critical, especially where criticism includes the payment of heavy fees to officials, and so hard to provide administrative ability of the highest kind.

